# Differences in serum markers of oxidative stress in well controlled and poorly controlled asthma in Sri Lankan children: a pilot study

**DOI:** 10.1186/s13223-020-00463-9

**Published:** 2020-07-22

**Authors:** Yenuli Fernando, Pujitha Wickramasinghe, Udani De Silva, Malintha Alahakoon, K. W. D. A. Anuradha, Shiroma Handunnetti

**Affiliations:** 1 Institute of Biochemistry, Molecular Biology and Biotechnology, University of Colombo, Colombo, Sri Lanka; 2 Colombo International School, Colombo, 7 Sri Lanka; 3 Department of Paediatrics, Faculty of Medicine, University of Colombo, Colombo, Sri Lanka; 4 Faculty of Medicine, University of Colombo, Colombo, Sri Lanka

**Keywords:** Childhood asthma, Oxidative stress, Nitric oxide, Antioxidants

## Abstract

**Background:**

Asthma is a disease characterised by hyper responsiveness and bronchoconstriction of airways, and is a major health burden globally. A dysfunction of the oxidant-antioxidant balance, termed oxidative stress, has been implicated in the pathophysiology of asthma. The present study aims to assess the changes in oxidative stress markers, namely nitric oxide metabolites and antioxidant capacity, in children with poorly controlled and well controlled asthma, in comparison to healthy controls.

**Methods:**

The present study enrolled 72 children (ages 5-15 years) classified into three groups: (1) poorly controlled asthma (n = 20), (2) well controlled asthma (n = 24) and (3) healthy controls (n = 27). An interviewer-administered questionnaire was used to record socio-demographic data of the participants. The serum concentrations of the oxidant markers (nitrite, nitrate and total nitric oxide metabolites [NO_x_]) were determined using the Griess test, and the total antioxidant capacity (TAOC) was determined using the ABTS decolorisation method. The concentrations of these markers were compared across the three groups.

**Results:**

The three study groups were similar in terms of socio-demographic data. The differences across the three groups were statistically significant for serum concentrations of nitrate and NO_x_ (but not nitrite) and serum TAOC. Further analyses showed that the disparity for nitrate and NO_x_ concentrations was greatest between poorly controlled asthma and healthy controls (p = 0.001 and p < 0.001) compared to the well-controlled asthmatics and healthy controls (p = 0.036 and p = 0.049). A significant difference in serum nitrate and NO_x_ concentrations was not observed between the two asthma groups (p = 0.311 and 0.203). The TAOC were significantly lower in poorly controlled asthmatics as compared to well-controlled asthmatics (p = 0.003) and healthy controls (p < 0.001). However, there was no significant difference in the serum TAOC between healthy controls and well-controlled asthmatics (p = 0.496). These findings may indicate that it is perhaps the higher TAOC that contributes to the well controlled state of asthma.

**Conclusions:**

The present study indicated that an imbalance of oxidants and antioxidants in the serum may have an underlying role in asthma pathophysiology, and how these markers may be effective in asthma management.

## Background

Asthma is a disease characterised by airway hyper-responsiveness, airway remodelling and reversible bronchoconstriction, affecting an estimated 235 million individuals worldwide, with a mortality of 383,000 deaths annually [1, 2]. The prevalence of asthma is rising in Sri Lanka where it occurs in 13–25% of children aged between 5 and 11 years, and is a major cause for school absenteeism in addition to being an economical burden in healthcare [3]. In 2015 alone, the Sri Lankan government allocated LKR 140 million (approximately 1 million CAD) for asthma treatment [4].

The full scope of molecular mechanisms underpinning the changes seen in asthma is still unclear. Plausible contributory mechanisms include genetic predisposition [5–9], epigenetic alterations [10, 11], exposure to environmental pollutants [12, 13], chemicals [14] and viral infections [15] acting as triggers of inflammation.

Recently, there has been an interest in the role of oxidant-antioxidant balance (oxidative stress) in the pathophysiology of asthma. Oxidative stress arises from an imbalance between the production of reactive oxygen species (ROS) and reactive nitrogen species (RNS) accompanied by a depletion of the antioxidant defense mechanisms [16]. RNS may be generated by the reactions of nitrite and nitric oxide (NO) with ROS [17]. The elevated production of NO, or ‘nitrosative stress’, may aggravate the detrimental effects of airway inflammation. Nitrite (NO_2_^−^) and nitrate (NO_3_^−^) are metabolites of NO oxidation by the superoxide anion, and serve as proxy measures of the unstable ROS [18]. Antioxidants include non-enzymatic molecules (glutathione, albumin, uric acid, bilirubin, vitamins A, C, and E, and several molecules/chemicals present in foods), and enzymatic systems such as superoxide dismutase (SOD), catalase and glutathione peroxidase [19]. Increased amounts of ROS and RNS in the blood diminish antioxidant capacity [20]. Oxidative stress has been implicated in many pathological conditions, including asthma [21–26]. As the severity of illness increases, antioxidants are depleted to a greater extent [27]. The persistent and intermittently exacerbated inflammation is accompanied by overproduction of ROS and RNS, which may also contribute to the promotion and persistence of the airway inflammation in asthma [23].

This pilot study aims to determine the markers of oxidative stress in children with poorly controlled (symptomatic) and well controlled asthma (asymptomatic) patients in comparison to an age-sex matched group of healthy controls, in order to determine if greater oxidative stress does occur in uncontrolled asthma. This could highlight the potential use of oxidative stress measurement as an indicator of disease severity and a plausible role of antioxidants in asthma management.

## Methods

### Clinical study design, sample size and data collection

This comparative study was carried out from June to October 2019, amongst children aged 5–15 years presenting to the outpatient clinic of the University Paediatric Unit of the Lady Ridgeway Children’s Hospital, which is the premier, tertiary level paediatric hospital in Sri Lanka. Three groups of children were enrolled: (1) poor asthma control while on corticosteroid/combined inhalers (according to Global Initiative for Asthma [GINA] criteria) (n = 20) (Table 1), (2) well controlled asthma for the past 12 months while on regular inhaled corticosteroid treatment (according to GINA criteria) (n = 24) and (3) age-sex matched healthy controls (n = 27).

**Table 1:** GINA assessment of asthma control in children 6-15 years and adolescents

Symptom control	Level of asthma symptom control			
In the past 4 weeks has the patient had		Well controlled	Partly controlled	Uncontrolled
Day symptoms more than twice a week	Y/N	None of these	1 to 2 of these	3 to 4 of these
Any night waking due to asthma	Y/N			
Relievers needed more than twice a week	Y/N			
Any activity limitation due to asthma	Y/N			

(Pocket guide for asthma management and prevention, GINA 2019)

The control group did not have any acute or chronic illness requiring long-term medications, atopic diseases or any symptoms of obstructive airway disease. Their peak expiratory flow rates were within the normal ranges for the age, height and sex. Age-sex matched children were recruited to all three groups when possible. Children with another comorbidity that can mimic asthma (e.g. congenital heart diseases, gastro-oesophageal reflux disease) as well as those without parental consent were excluded.

There have been no previous studies that had measured oxidative stress in asthma in Sri Lanka. Hence the sample size was calculated based on an analogous study that assessed oxidative stress in fever patients in Sri Lanka using the same assays [28]. The mean difference and standard deviations of nitric oxide metabolites between healthy controls and fever patients in the aforementioned study was used as guidance to estimate sample size with a power of 80% and a 95% confidence interval for this study, which indicated that the sample size should at least be 20 per group. A convenient sampling technique with case matching was used.

An interviewer-administered questionnaire was used to record socio-demographic data (including monthly family income and parents level of education), and to clarify the degree of asthma control focussing on the GINA criteria. A general health check up was carried out for all children after consenting to participate. A stool smear examination, Full Blood Count and ESR was done free of charge. No parasites or parasitic stages were found on examination of saline and iodine smears. There was no significant difference in the ESR levels between the asthmatic children and healthy children.

### Sample collection, transport and storage

Trained experienced nurses in the clinic collected blood under aseptic conditions. Since serum nitrate is dependent on dietary factors [29–32], blood samples were collected 8 h after the last meal in the morning. 3 ml of blood was put into a sterile plain tube and allowed to clot for 15 min at room temperature prior to centrifugation at 2000 rpm for 15 minutes [33]. Serum was separated and stored at − 20 °C until analysis was carried out for estimation of oxidative stress markers.

### Assays to determine the nitrite, nitrate and NO_x_ concentrations and antioxidant capacity

All laboratory tests were carried according to previously published protocols [34, 35].

In brief, the Griess reaction was used to determine the concentrations of nitrite and total nitric oxide metabolites (NO_x_ denoting NO_2_^−^ and NO_3_^−^) in test sera. The sera were deproteinised chemically by adding 1.5 g/ml zinc sulfate and mixing for 1 min (by vortex). The mixtures were centrifuged at 10 000 g for 15 min, and the supernatant was separated and centrifuged at 10 000 g for 10 min. The serum nitrite concentration was analysed by mixing 100 μl of deproteinised serum and 100 μl of Griess reagent (prepared as a 1:1 mixture of 1% sulphanilamide in 5% phosphoric acid and 0.1% N-(1-naphytyl) ethylenediamine hydrochloride) followed by an incubation of 15 min in dark at room temperature. The serum NO_x_ concentration was analysed by mixing 70 μl of deproteinised serum, 70 μl of 8 mg/ml vanadium (III) chloride and 70 μl of Griess reagent, followed by an incubation of 30 min at room temperature in dark.

The absorbance was measured using a spectrophotometer (Synergy HT multimode microplate reader, Biotek, USA) at 540 nm in 96-well microplates in duplicates alongside series of sodium nitrite two-fold dilutions (100 μM–0.195 μM) and reagent blanks. The concentrations of nitrite and NO_x_ in sera were calculated using a standard curve made from serial dilutions. As the serum NO_x_ represents nitrite and nitrate in serum, the serum nitrate concentration was determined according to Fitzpatrick et al. by deducting the serum nitrite concentration from the serum NO_x_ concentration [18].

The total antioxidant capacity (TAOC) of test sera was analyzed using the 2,2′-azinobis-(3-ethylbenzothiazoline-6-sulfonic acid) (ABTS) decolourisation method. To facilitate the formation of radical cations (ABTS•^+^) by oxidation, 7 mM ABTS solution and 2.4 mM potassium persulphate (K_2_S_2_O_8_) were mixed in equal volumes and maintained in dark for 5 h. The ABTS working solution was prepared by diluting the mixture with 5 mM phosphate/0.145 M NaCl pH 7.4 phosphate buffered saline (PBS) according to the dilution factor which produces an optical density of ≈0.400 at 734 nm (determined using an ABTS:PBS dilution series). The test sera were analyzed in 96-well microplates in duplicates alongside a series of 6-Hydroxy-2,5,7,8-tetramethyl-chroman-2-carboxylic acid (Trolox) two-fold dilutions (400 μM–5 μM), reagent blanks (a mixture of 10 μl of distilled water, 10 μl of K_2_S_2_O_8_ and the volume of PBS that is the product of 20 μl and the ABTS:PBS dilution factor) and controls (a 1:19 mixture of PBS and working ABTS solution).

Test sera were mixed with ABTS working solution in a 1:19 ratio within a minute in dark. The absorbance at 734 nm was determined using a spectrophotometer (Synergy HT multimode microplate reader, Biotek, USA) at 734 nm. The Trolox standard curve was used to determine the serum TAOC as expressed as a Trolox equivalent antioxidant capacity (TEAC).

### Data analysis

The data were transferred into a Microsoft Excel database and checked for accuracy. The analysis was done using Statistical Package for the Social Sciences/Statistical Product and Service Solutions (SPSS) version 23.0 (IBM, USA). Descriptive statistics were summarised with measures of central tendency and measures of dispersion. Discrete variables across study groups were compared with Chi square test. The laboratory assay results were arranged into data arrays, tested for normal distribution and differences across groups were tested with the Kruskal–Wallis test, Mann–Whitney U test and Spearman’s correlation as appropriate. A p value < 0.05 was considered as statistically significant.

## Results

### Characteristics of the study population

A total of 72 children were enrolled in the study; 20 children with poorly controlled asthma, 25 children with well controlled asthma and 27 healthy children.

When the baseline socio-demographic characteristics in the three groups of children were considered, there was a similar age and gender distribution across all three groups. The mean age (expressed as mean ± standard deviation in years) of children with well controlled asthma, poorly controlled asthma and controls was 8.96 ± 2.58, 8.80 ± 2.52 and 9.97 ± 2.82, respectively. There were no significant differences in the socio-demographic characteristics, namely mother’s and father’s education and monthly family income between the three groups (p > 0.05). Considering differences in co-morbidities between poorly and well controlled asthmatic children, except for higher number of allergic rhinitis in former, there were no significant difference in food or drug allergy, eczema or obesity.

### Markers of oxidative stress across the study groups

The mean serum nitrite, nitrate and NO_x_ concentrations were highest in the poorly controlled asthma group and lowest in the healthy controls. The mean serum nitrite, nitrate and NO_x_ concentrations in the well-controlled asthma group was lower than in the poorly controlled asthma group, but higher than the healthy controls. In contrast, the highest mean serum TAOC was observed in the healthy controls group while the lowest mean serum TAOC was observed in the poorly controlled asthma group (Table 2).

**Table 2:** Comparison of mean (± standard deviation) of test parameters between the study groups

	Poorly controlled asthma (n = 20)	Well controlled asthma (n = 24)	Healthy controls (n = 27)
Serum nitrite concentration (μM)	0.36 ± 0.65	0.17 ± 0.20	0.13 ± 0.18
Serum nitrate concentration (μM)	5.02 ± 2.02	4.46 ± 2.12	3.19 ± 1.16
Serum NO_x_ concentration (μM)^a^	5.37 ± 2.12	4.63 ± 2.19	3.32 ± 1.21
Serum TAOC (μM)	336.40 ± 20.38	359.33 ± 25.46	367.12 ± 12.45

^a^NO_x_ denotes NO_2_^−^ and NO_3_^−^ collectively

**Fig. 1:**
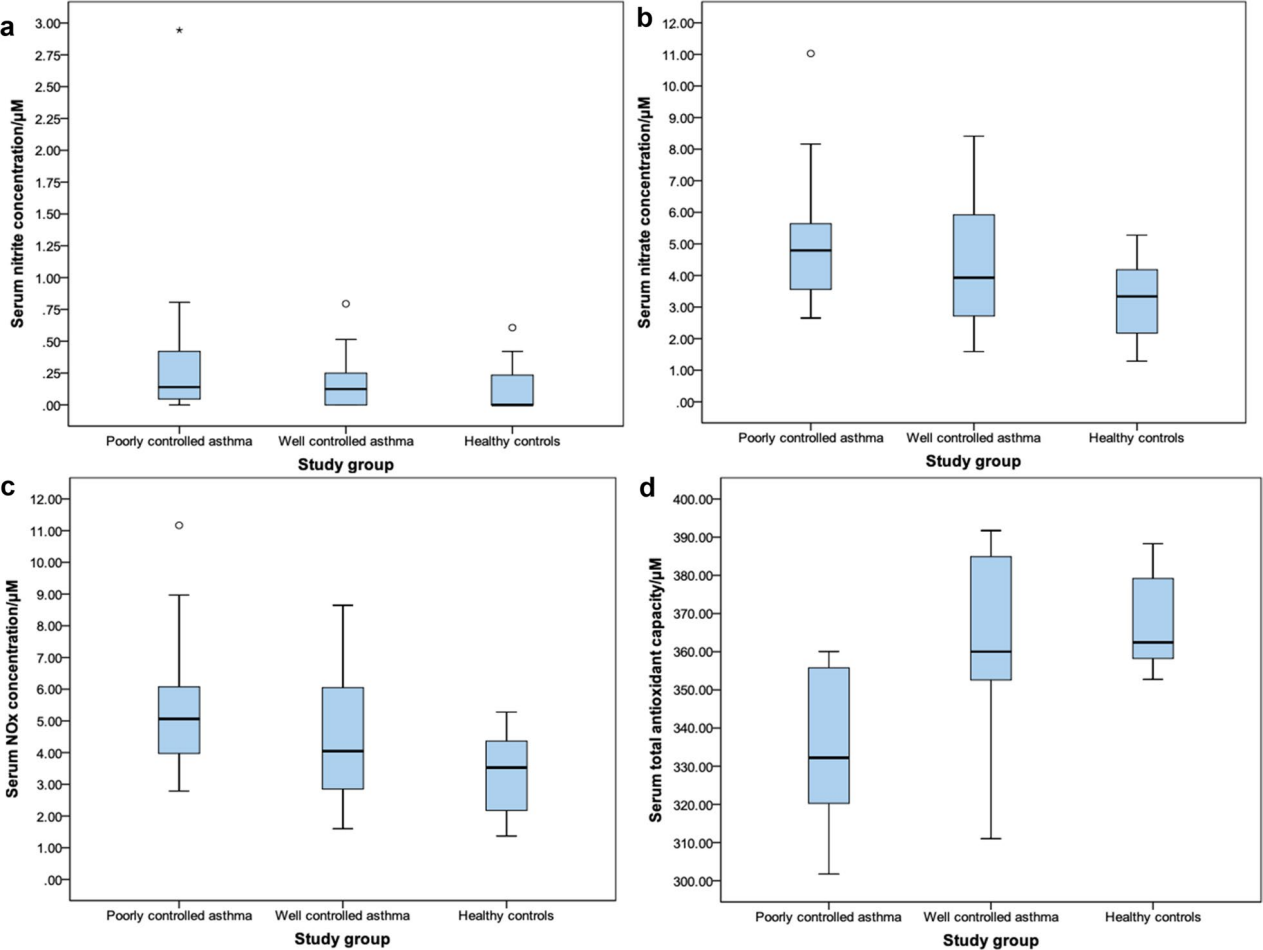
Boxplots of serum concentrations of (**a**) nitrite, (**b**) nitrate and (**c**) NO_x_ and (**d**) serum TAOC

When the medians were considered, there were significant differences across the study groups with regard to serum nitrate and NO_x_ concentrations (but not nitrite concentrations) and serum TAOC (Table 3 and Fig. 1).

**Table 3:** Comparison of medians (± interquartile range) of test parameters between the study groups

	Poorly controlled asthma (n = 20)	Well controlled asthma (n = 24)	Healthy controls (n = 27)	p value
Serum nitrite concentration (μM)	0.14 ± 0.37	0.12 ± 0.26	0 ± 0.23	0.149
Serum nitrate concentration (μM)	4.79 ± 2.15	3.93 ± 3.28	3.34 ± 2.15	0.003*
Serum NO_x_ concentration (μM)^a^	5.06 ± 2.31	4.05 ± 3.32	3.53 ± 2.35	0.003*
Serum TAOC (μM)	333.20 ± 36.39	360.02 ± 34.10	362.45 ± 12.45	< 0.001*

^a^NO_x_ denotes NO_2_^−^ and NO_3_^−^ collectively

*p < 0.05 comparison between the three study groups (Kruskal–Wallis Test)

Further analysis of significant variables across the groups using the Mann–Whitney U Test showed that the disparity between groups for nitrate and NO_x_ concentrations was greatest between the poorly controlled asthma and healthy controls groups (p = 0.001 and p < 0.001 respectively) compared to the well-controlled asthma and healthy controls groups (p = 0.036 and p = 0.049 respectively). There were no significant differences in these parameters between the well-controlled asthma and the poorly controlled asthma groups (p = 0.311 and 0.203 respectively). The Mann–Whitney U Test analyses showed that the serum TAOCs were significantly different across the two asthma groups (p = 0.003) and across the poorly controlled asthma group and healthy controls (p < 0.001). There was no statistically significant difference between the well-controlled asthma and healthy controls groups (p = 0.496) (Table 4).

**Table 4:** Comparison of medians between the groups using the Mann–Whitney U test

	Poorly controlled asthma (n = 20)	Well controlled asthma (n = 24)	Healthy controls (n = 27)	p-value
Serum nitrate concentration/μM	4.79 ± 2.15	3.93 ± 3.28		0.311
Serum NO_x_ concentration/μM^a^	5.06 ± 2.31	4.05 ± 3.32		0.203
Serum nitrate concentration/μM		3.93 ± 3.28	3.34 ± 2.15	0.036*
Serum NO_x_ concentration/μM^a^		4.05 ± 3.32	3.53 ± 2.35	0.049*
Serum nitrate concentration/μM	4.79 ± 2.15		3.34 ± 2.15	0.001*
Serum NO_x_ concentration/μM^a^	5.06 ± 2.31		3.53 ± 2.35	< 0.001*
Serum TAOC/μM	333.20 ± 36.39	360.02 ± 34.10		0.003*
Serum TAOC/μM		360.02 ± 34.10	362.45 ± 12.45	0.496
Serum TAOC/μM	333.20 ± 36.39		362.45 ± 12.45	< 0.001*

^a^NO_x_ denotes NO_2_^−^ and NO_3_^−^ collectively

*p < 0.05 comparison between the three study groups (Mann–Whitney U test)

### Correlation of oxidants and antioxidant capacity

The serum TAOCs of the study population were correlated with the respective serum nitrite, nitrate and NO_x_ concentrations in order to determine how the antioxidants in the serum may change due to the increased generation of the oxidants.

**Fig. 2:**
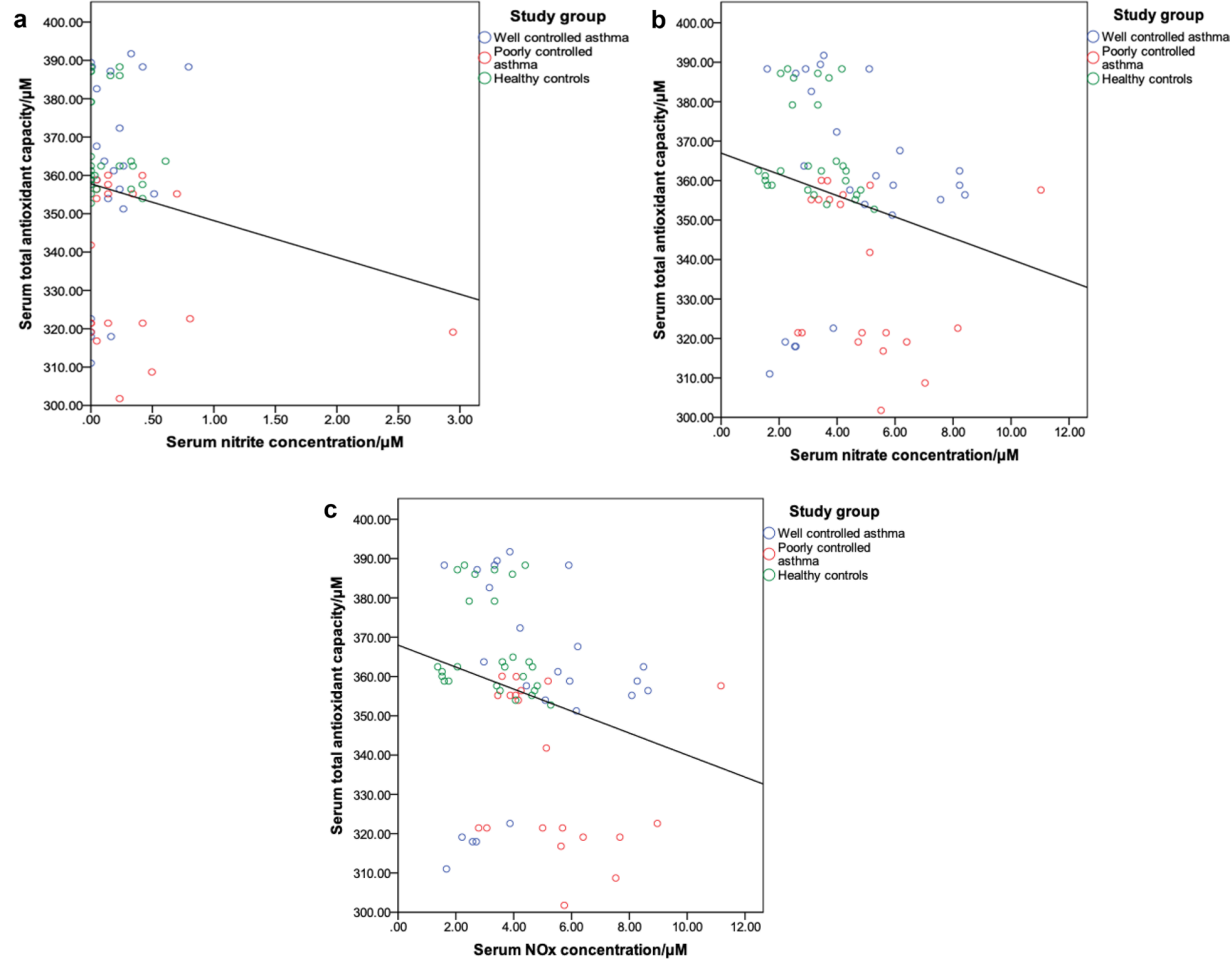
Scatterplots for the correlation between serum TAOCs and (**a**) serum nitrite concentration (r = − 0.006, p = 0.959), **b** serum nitrate concentration (r = − 0.306, p = 0.010) and (**c**) serum NOx concentration (r = − 0.312, p = 0.008). The Spearman’s correlations between the serum TAOC and the serum nitrate and NOx concentrations are statistically significant

Using the Spearman correlation, significant negative correlations were shown only for the serum TAOCs between the serum nitrate (r = − 0.306, p = 0.010) and NO_x_ (r = − 0.312, p = 0.008) concentrations at the 0.01 level (Fig. 2).

## Discussion

The present study is the first in Sri Lanka to measure the biomarkers of oxidative stress in patients with asthma. The results have clearly demonstrated that the concentrations of serum nitrate and NO_x_, two biomarkers for oxidative stress, were significantly elevated in patients with asthma regardless of the level of disease control compared to healthy, non-asthmatic children. Conversely, serum TAOC was highest in healthy controls, and this was statistically significantly different from that of patients with poorly controlled asthma. There was no significant difference between healthy controls and well-controlled asthmatics with regard to serum TAOC.

Oxidative stress can be a manifestation of inflammation as well as an initiator of inflammation [36]. There are different biomarkers to assess the redox status of tissues including free radicals, lipid peroxidation products and alterations of antioxidant levels (as a surrogate marker). In this study serum nitrites, nitrates and TAOC were used as these assays have been used and validated for the local population previously for non-asthma related pathologies including leptospirosis [28, 35, 37], dengue haemorrhagic fever [34] and Parkinson disease [38]. Other studies which measured pro-oxidant levels in patients with asthma used exhaled nitric oxide (eNO) as this is a sensitive and specific marker of airway inflammation and a higher level of eNO was recorded in patients with asthma as compared to healthy controls [39–41]. However, routine measurements of eNO are quite costly to be implemented in the resource-limited settings of developing countries such as Sri Lanka, and in addition to that the molecule itself is volatile. Further, it is comparatively more difficult to measure the eNO levels in a paediatric population [42]. Using bronchoalveolar lavage as a source of secretions and airway cells would have been better but requires an invasive procedure, which can only be used in stable, well-controlled asthma. Another option is to assess the NO concentration in sputum but the levels may vary depending on whether the sputum is induced or not, and once again sputum is difficult to collect in children [43].

In this study, the approximate cost of carrying out serum NO levels was estimated at 3 CAD per sample. Thus the current study has demonstrated that measurement of serum NO may be a more cost-effective and practical approach in paediatric populations to demonstrate the occurrence of an underlying pathology in asthmatics as compared to a healthy population taking into consideration that measurement of eNO levels costs approximately 32 CAD per test [44, 45]. The serum nitrite concentration did not show any significant differences across the study groups, while the serum nitrate concentration, which is obtained by subtracting nitrite concentration from NOx concentration, did show a statistically significant difference across the groups. Hence it can be argued that measurements of the serum NO concentrations with the modified Griess method alone may be adequate to differentiate oxidative stress between these groups. The difference in total NO concentration may manifest as a change in both nitrite and nitrate levels but since the latter anion is more stable, it is perhaps more accurately quantified than the nitrite level [46]. It is also known that approximately all of the nitrite in the systemic circulation is converted to nitrate by oxyhaemoglobulin during the l-arginine-NO pathway [47]. The total serum NO concentration was significantly higher in all asthmatics compared to healthy controls. This probably indicates the same intensity of inflammatory activity in airways of all asthmatics regardless of whether it is well controlled or not (there was no significant difference between well controlled and poorly controlled asthmatics).

On the contrary, as demonstrated in other studies [39, 48–50], the TAOC in this study was also significantly higher in well-controlled asthmatics and healthy children compared to poorly controlled asthmatics. Hence the antioxidant capacity may have a significant contribution to the clinical phenotype of “well controlled asthma” despite a same degree of oxidative stress. Furthermore, as antioxidants scavenge the surplus free radicals to reduce inflammation, the potential of antioxidants in the prevention and treatment of diseases has been studied in recent years [51]. Nadeem et al. [49], studied the oxidant- antioxidant imbalance in 38 patients with bronchial asthma and 23 control subjects in India. This study concluded that as a reduction in the antioxidant defenses (SOD enzyme) was associated with tissue damage in asthma, therapeutic augmentation of the anti-oxidant defences may be beneficial [49]. For example, in a NO-rich environment, SOD enzyme was shown to be less active leading to ROS and RNS generation [25, 52]. The cumulative effect of SOD inactivation and oxidative stress led to apoptosis of airway cells, and perhaps contributed to airway hyperresponsiveness in asthma.

The beneficial effects of consuming an antioxidant-rich diet have also been studied [52, 53]. One of these studies showed that a deficiency in vitamin C, which is the predominant antioxidant in the airway surface liquid, is associated with the elevated oxidative stress in asthma [52]. In addition, effective treatment of asthma may also help to build up the antioxidant reserves in asthmatics. Using antioxidants as therapeutics is a popular topic in academia and alternative treatment advocates. While the beneficial effects of such interventions were not tested, this study demonstrated that effective treatment for asthma itself could boost the antioxidant reserve in serum quite significantly compared to poorly controlled patients. As asthma therapy, which is managed in a step-manner, is well established, evidence-based, cheap and usually successful (provided that compliance and technique is good), this should be the primary and preferred method of asthma control over unproven methods such as dietary supplementation with antioxidants. This study has given an insight into the potential use of measurement of oxidative stress as an indicator of disease severity and, whether the use of antioxidants may be implemented as a therapeutic measure in disease management.

This study had some limitations. Even though healthy children with no apparent acute illness were selected, there is a possibility that the children suffered from some sort of acute illness in the recent past. The assays were done on serum biomarkers which may have been influenced by pathologies other than asthma (although the necessary precautions were taken to include children who had no other known co-morbidities at the time of recruitment for the study). The study assessed the plasma values of NO but as the primary pathophysiology is in the airway it would have been better to sample the airway NO levels. A study by Morris et al. [54], showed that eNO levels were significantly higher in asthma patients compared to healthy individuals although no significant difference was demonstrated for the NO metabolites in serum. Further studies will also be required to determine whether the higher differences in biomarkers is a by-product of on going inflammation due to asthma or whether the changes in the biomarkers actually contribute to the worsening of asthma (chicken and egg phenomenon). It is not clear whether the well-controlled asthmatics/healthy controls have improved antioxidant or whether increased antioxidants have improved the health status. Due to financial restrictions, the study was carried out during a specific time period (June to November 2019) thus the sample size was limited to the minimum number required to show an effect. The study participants were instructed to maintain an 8-h window between their last meal and sample collection due to the profound influence dietary nitrate can have on the plasma nitrite and nitrate levels; however, it cannot be certain that the participants adhered to the restriction.

## Conclusions

As hypothesised, a significant increase in serum NOx concentration was observed in children with asthma (compared to healthy controls) regardless of whether the asthma is well controlled or not. Similarly, Healthy controls and those who had well-controlled asthma had significantly higher TAOC of serum compared to poorly controlled asthma patients.

It can be concluded that serum TAOC assessment has potential to be developed as a cheap, easy to perform assay to objectively confirm good asthma control. However, these findings need to be verified with a larger prospective study with accurate recording of clinical exacerbations (and not just by history taking) enabling control of multiple confounding factors.

## Data Availability

The data and material is available with the Principal Investigator.

## Abbreviations

ABTS: 2,2′-azinobis: (3-ethylbenzothiazoline-6-sulfonic acid)
BMI: Body Mass Index
eNO: Exhaled Nitric Oxide
GINA: Global Initiative for Asthma
IBMBB: Institute of Molecular Biology, Biochemistry and Biotechnology
LKR: Sri Lankan Rupees
NO: Nitric oxide
NO_x_: Nitric oxide metabolites
NO_2_^−^: Nitrite
NO_3_^−^: Nitrate
PBS: Phosphate buffered saline
ROS: reactive oxygen species
RNS: Reactive nitrogen species
SOD: Superoxide dismutase
TAOC: Total antioxidant capacity
TEAC: Trolox equivalent antioxidant capacity
WHO: World Health Organisation
